# The Carbonic Anhydrase βCA1 Functions in PopW-Mediated Plant Defense Responses in Tomato

**DOI:** 10.3390/ijms241311021

**Published:** 2023-07-03

**Authors:** Jieru Zhao, Zhixiang Yuan, Xixi Han, Tingting Bao, Tingmi Yang, Zhuang Liu, Hongxia Liu

**Affiliations:** 1 Department of Plant Pathology, College of Plant Protection, Nanjing Agricultural University, Nanjing 210095, China; 2020102020@stu.njau.edu.cn (J.Z.); 15950387417@163.com (Z.Y.);; 2 Guangxi Academy of Specialty Crops, Guilin 541004, China

**Keywords:** carbonic anhydrase, Harpin protein, plant immunity, PopW, protein interaction, *Solanum lycopersicum*, *Xanthomonas euvesicatoria*

## Abstract

β-Carbonic anhydrase (βCA) is very important for plant growth and development, but its function in immunity has also been examined. In this study, we found that the expression level of *Solanum lycopersicum βCA1* (*SlβCA1*) was significantly upregulated in plants treated with *Xanthomonas euvesicatoria* 85-10. The protein was localized in the nucleus, cell membrane and chloroplast. Using tomato plants silenced with *SlβCA1*, we demonstrated that SlβCA1 plays an active role in plant disease resistance. Moreover, we found that the elicitor PopW upregulated the expression of *SlβCA1*, while the microbe-associated molecular pattern response induced by PopW was inhibited in TRV-*SlβCA1*. The interaction between PopW and SlβCA1 was confirmed. Here, we found that SlβCA1 was positively regulated during PopW-induced resistance to *Xanthomonas euvesicatoria* 85-10. These data indicate the importance of SlβCA1 in plant basic immunity and its recognition by the Harpin protein PopW as a new target for elicitor recognition.

## 1. Introduction

Plant diseases are considered one of the most important factors threatening plant growth and yield [1]. Plants have evolved two lines of defense to resist invasion by various pathogenic microorganisms [2]. The two lines of defense that have evolved in plants include pattern recognition receptor (PRR)-triggered immunity (PTI) induced by pattern PRRs on the cell surface and effector-triggered immunity (ETI) induced by nucleotide-binding/leucine-rich-repeat receptor (NLR) recognition in the cell [3]. In host plants, recognition-receptor-triggered immunity effectively repels most pathogens while benefiting basic immunity [3]. Therefore, the discovery of the receptors involved in the basic plant immune response is very important for studying the molecular mechanism underlying plant disease resistance.

Carbonic anhydrase (CA), a zinc-containing metalloenzyme, is a CO_2_-binding protein that enzymatically catalyzes the interconversion of CO_2_ and HCO_3_^−^ [4,5]. In animals, CA is closely related to pathological metabolic pathways, including lipogenesis, gluconeogenesis, ureogenesis, tumor formation and hypercapnia pathways [6,7]. The CA protein content in higher plants can account for 1–2% of the soluble leaf protein content [8]. During the long process of biological evolution, a variety of CA families with different structures have emerged. Plant CAs include three subtypes, α, β and γ, which have no significant sequence homology and evolved independently [5,9]. The organs in which the three subtypes of CA proteins are expressed also differ. The αCA and γCA proteins are highly expressed in reproductive organs, so they may play a role in developmental regulation, while the βCA proteins are mainly expressed in plant leaves [9].

The relationship between the function of βCA and photosynthesis is a major research direction [9]. Several findings suggest that βCA, in addition to being involved in photosynthesis, is also involved in plant defense against pathogens [10]. First, some reports have shown that CA is differentially expressed at the transcriptional or protein level during pathogen infection, suggesting its potential role in plant defense [10]. In 2005, Restrepo et al. found by cDNA chip technology that the expression of *βCA* was inhibited after *Phytophthora infestans* infection [11]. Additionally, the expression of the *β*CA gene was suppressed by infection with *Plasmopara viticola* [12]. In contrast, five βCA proteins have been shown to be involved in the infection of non-heading Chinese cabbage with the downy mildew *Hyaloperonospora parasitica* by proteomic analysis [13]. Moreover, there is evidence that βCA can play a role in plant immunity. In *Nicotiana benthamiana*, the suppression of *βCA* expression by genetic manipulation increases host susceptibility to *P. infestans* [11]. In Arabidopsis, βCA1 and βCA4 can reduce resistance to *Pseudomonas syringae* pv. *tomato* (*Pst*) DC3000 [10]. Although the above evidence shows that βCA can enhance plant defense against disease, this is not always the case. Wang et al. reported that AtβCA1 can negatively regulate Arabidopsis immunity to *Pst* DC3000 [14]. In addition, βCA has been reported to participate in immune processes, including intracellular and cell surface recognition-receptor-triggered immunity. In *N. benthamiana*, *βCA1* can inhibit the leaf-hypersensitive response induced by avrPto [15]. Salicylic acid (SA)-binding protein 3 (SABP3), also known as NbβCA1 [15], binds to SA in vitro. Zhou et al. showed that βCA1 and βCA4 inhibited the PTI induced by flg22 [10].

Although there is much evidence showing that βCA plays an important role in plant immunity, research on the specific resistance mechanism and molecular interaction mechanism of βCA is still in the early stage. A study showed that the *βca1βca4* double mutant and the *βca1βca4βca6* triple mutant were not sensitive to the changes in stomatal conductance induced by CO_2_ [16]. Hu et al. demonstrated that AtβCA1 and AtβCA4 are upstream regulators of stomatal movement in guard cells [17]. Notably, stomatal closure is a key part of the plant immune response, and stomata are also an invasion point for many leaf pathogens [18]. The Arabidopsis resistance to *Pst* DC3000 was not damaged in the *βca1βca2βca3βca4βca6* mutant, but effector-triggered immunity was impaired in the quintuple mutant [16]. Recently, several βCAs have been shown to play a role in SA sensing in Arabidopsis. AtβCA1 can interact directly with AtNPR1, and AtβCAs can also interact with NRB4 [16]. Wang et al. found that the AtβCA1 protein could undergo S-nitrosylation, which further affected the SA binding ability and CA activity of the protein and negatively regulated immunity [14]. However, some studies have shown that βCA3 can participate in the positive regulation of basic immunity against *Pst* DC3000, and the regulatory pathway is independent of stomatal and SA-dependent regulation [19]. The immune function of other β-CAs is unknown.

PopW, as a biological elicitor of the Harpin protein, was originally isolated from *Ralstonia solanacearum* (*Rs*) ZJ3721 [20]. PopW can improve plant immunity. At present, the diseases that PopW can confer resistance to include tomato leaf mold, tomato bacterial scab, tomato bacterial wilt, cucumber downy mildew, tobacco mosaic virus disease and rice false smut [21,22,23,24]. It was also found that PopW could stimulate the PTI response of tomato to confer resistance to *Xanthomonas euvesicatoria* (*Xe*) 85-10 infection, and the resistance mechanism was related to stomatal immunity [24]. PopW can not only stimulate resistance to various diseases in plants but also promote plant growth and improve plant stress resistance [22,23,25].

Given the response of the *SlβCA1* gene after *Xe* 85-10 inoculation, we chose to study the function in basal immunity of SlβCA1 in the model plant tomato. We provide evidence that inhibition of *SlβCA1* gene expression attenuates plant defense responses to *Xe* 85-10 infection. In addition, it was previously reported that βCA could act as a negative regulator of the elicitor-induced microbe-associated molecular pattern (MAMP) response. Therefore, we selected an elicitor, PopW, which can enhance tomato defense against *Xe* 85-10, to determine whether SlβCA1 can affect the MAMP response. The results showed that SlβCA1 can upregulate the PopW-induced MAMP response and directly interact with PopW, thus reducing PopW-induced resistance to *Xe* 85-10. The immune function of SlβCA1 is not related to CA activity. These findings provide insights into the role of βCA and PopW in plant immunity.

## 2. Results

### 2.1. SlβCA1 Expression Was Induced by Pathogenic Bacteria

**Figure 1:**
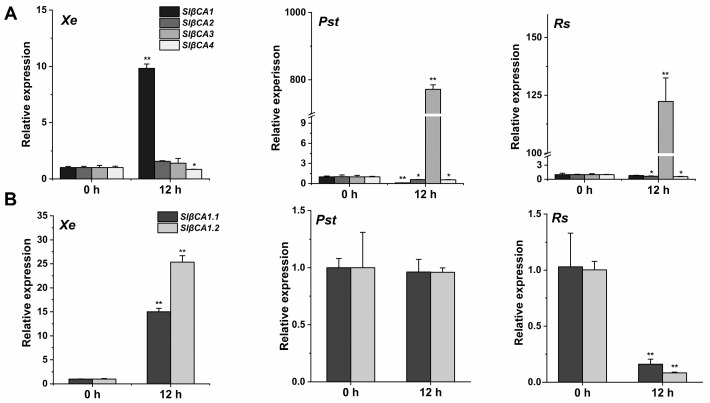
*Xanthomonas euvesicatoria* (*Xe*) 85-10 upregulated SlβCA1 expression. (**A**) In leaves of 6 weeks old, *SlβCA1*, *SlβCA2*, *SlβCA3* and *SlβCA4* expression levels at 0 and 12 h after spray inoculation with *Xe* 85-10, *Pseudomonas syringae* pv. *Tomato* (*Pst*) DC3000 and *Ralstonia solanacearum* (*Rs*) ZJ3721. The gene amount in the 0 h (untreated) sample was assigned a numerical value of 1. (**B**) Transcript abundance of *SlβCA1* in response to *Xe* 85-10, *Pst* DC3000 and *Rs* ZJ3721 at 0 h and 12 h. The gene amount in the 0 h (untreated) sample was assigned the numerical value of 1. The asterisks indicate statistical significance by *t* test (** *p* < 0.01; * *p* < 0.05).

Reportedly, the transcription of *βCA*s exhibits different responses to infection by various pathogens in different plants [10]. All six *AtβCAs* can respond to pathogen infection, among which *AtβCA1*, *AtβCA2*, *AtβCA4* and *AtβCA5* generally show reduced expression in response to infection with 16 plant pathogens, while *AtβCA3* is significantly upregulated after treatment with *P. syringae*, *Alternaria brassicicola*, *Rhizoctonia solani*, *Sclerotinia sclerotiorum* and *Golovinomyces cichoracarum*, and *AtCA6* is significantly upregulated after infection with *P. infestans* and *Myzus persicae* [10]. In tomato, Hu et al. identified four SlβCAs based on amino acid sequence similarity [19]. In their study, *SlβCA3* showed high expression after *Pst* DC3000 treatment [19]. Consistently, our results showed significant upregulation of *SlβCA3* after *Pst* DC3000 and *Rs* ZJ3721 treatment (Figure 1A) [19]. Interestingly, only *βCA1* was highly expressed in response to *Xe* 85-10 inoculation; transcription levels of *β*CA2, *βCA3* and *βCA4* were unaltered or decreased in response to *Xe* 85-10 inoculation (Figure 1A).

The National Center for Biotechnology Information database contains two transcripts of *SlβCA1*. We monitored the transcription of *SlβCA1.1* and *SlβCA1.2* after treatment with three pathogens. After treatment for 12 h, *SlβCA1.1* and *SlβCA1.2* were both highly expressed in response to *Xe* 85-10. The transcription levels of *SlβCA1.1* and *SlβCA1.2* were unchanged or decreased after *Pst* DC3000 or *Rs* ZJ3721 treatment (Figure 1B). Therefore, in the following experiments, we used SlβCA1.

### 2.2. Subcellular Localization and Expression Pattern of SlβCA1

**Figure 2:**
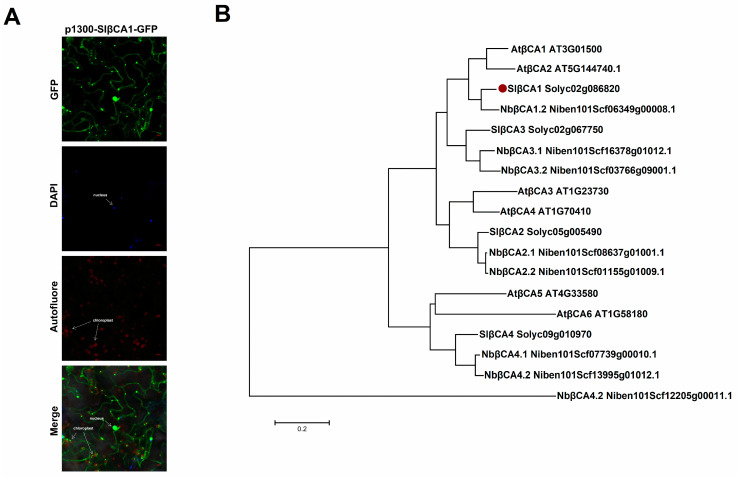
Localization, tissue expression specificity and evolutionary relationship of SlβCA1. (**A**) Subcellular localization of SlβCA1 expression was observed using confocal microscopy in tobacco leaves. Green, GFP signal; red, chloroplast autofluorescence indicating the locations of chloroplasts; blue, DAPI signal indicating the locations of nuclei. Scale bar = 20 μm. (**B**) Phylogenetic tree of βCA isoforms from *tomato*, *N. benthamiana* and *Arabidopsis thaliana*. SlβCA1 are highlighted using red dot.

We expressed green fluorescent protein (GFP)-SlβCA1 in *N. benthamiana* cells to determine the subcellular localization of SlβCA1. The GFP signal observed in tobacco cells indicated that SlβCA1 was localized in the nucleus and chloroplast and near the cell membrane (Figure 2A).

Amino acid sequence alignment of the tomato, Arabidopsis and tobacco proteins revealed a close relationship between SlβCA1 and NbβCA1.2 (Figure 2B). These results suggest that SlβCA1 may have a potential functional relationship with NbβCAs; NbβCAs have been reported to be involved in the positive regulation of plant immunity [11,15].

### 2.3. SlβCA1 Is Involved in the Positive Regulation of Immunity against Xe 85-10

**Figure 3:**
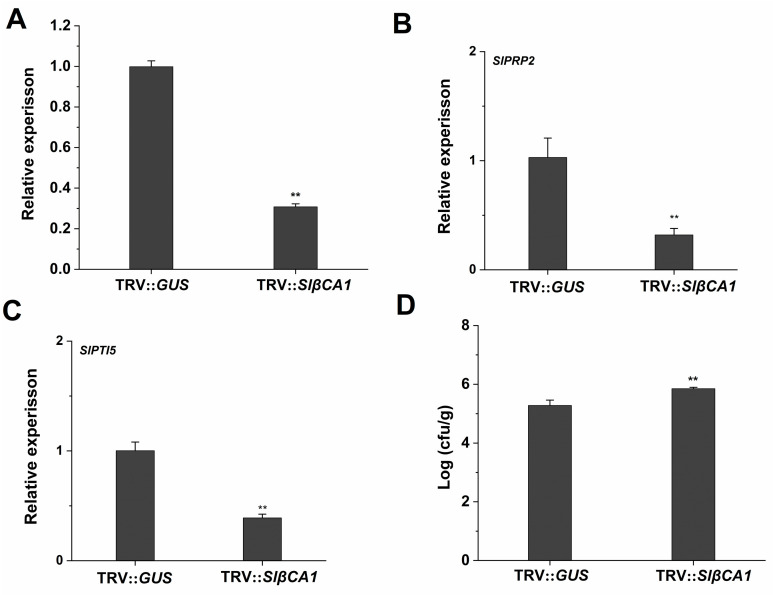
SlβCA1 positively regulates immunity to *Xe* 85-10. (**A**) Expression level of *SlβCA1* in 6-week-old leaf samples of TRV-*SlβCA1* and TRV-*GUS*. The amount of *SlβCA1* in the TRV-*GUS* sample was assigned the numerical value of 1. (**B**,**C**) Expression levels of *SlPRP2* and *SlPTI5* in tomato leaves after 24 h of *Xe* 85-10 spraying treatment. The amount of the gene in the TRV-*GUS* sample was assigned a numerical value of 1. (**D**) Bacterial growth after 4 d of spraying treatment. The asterisks indicate statistical significance by *t* test (** *p* < 0.01).

To study the role of SlβCA1 in the response to pathogen infection, we silenced *SlβCA1* in tomato (TRV-*SlβCA1*) and used tomato with *glucuronidase* silenced (TRV-*GUS*) as a negative control (Figure 3A). The expression level of the resistance-related genes *SlPR-P2* and *SlPTI5* in TRV-*SlβCA1* was significantly reduced compared to that in TRV-*GUS* in response to pathogens, when this response was measured 24 h after *Xe* 85-10. These results suggest that SlβCA1 may be involved in plant immunity to *Xe* 85-10 (Figure 3B,C).

To further explore the immune function of SlβCA1 during *Xe* 85-10 infection, the colonization amount of the pathogenic bacteria in the leaves was detected on the second day after inoculation. The results showed that compared to a negative control, the bacterial colonization amount was significantly higher on the fourth day in TRV-*SlβCA1* (Figure 3D).

### 2.4. SlβCA1 Expression Was Induced by MAMP

**Figure 4:**
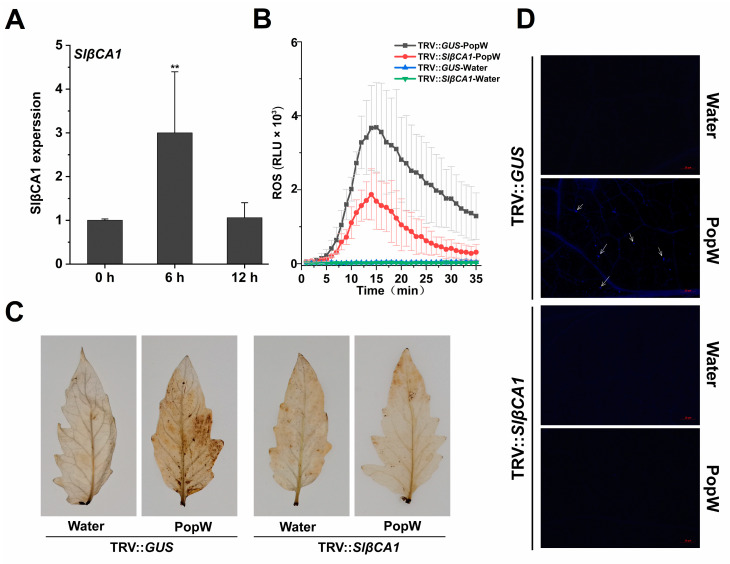
SlβCA1 enhanced the MAMP response of PopW. (**A**) *SlβCA1* expression levels of the WT at 0 h, 6 h and 12 h after PopW (1 μM) treatment. The amount of *SlβCA1* in the 0 h (untreated) sample was assigned the numerical value of 1. (**B**) ROS burst induced by PopW (10 μM) or water treatment in leaf discs of TRV-*GUS* and TRV-*SlβCA1*. (**C**) ROS accumulation induced after PopW (1 μM) or water treatment for 3 h in leaves of TRV-*GUS* and TRV-*SlβCA1*. (**D**) Callose induced after PopW (1 μM) or water treatment for 12 h in leaves of TRV-*GUS* and TRV-*SlβCA1*. Scale bar = 20 μm. The asterisks indicate statistical significance by *t* test (** *p* < 0.01).

Next, we tested whether the induction of *SlβCA1* expression by pathogens could be a MAMP-induced response. To this end, we examined the expression pattern of *SlβCA1* after treatment with PopW, an elicitor (pathogen signal metabolite) derived from *Rs* ZJ3721. As shown, after PopW treatment, *SlβCA1* expression was significantly induced at 6 h (Figure 4A).

We also examined the downstream PTI responses triggered by MAMPs, including the burst of reactive oxygen species (ROS), the accumulation of ROS and the accumulation of callose. Silencing of *SlβCA1* significantly attenuated the PopW-induced ROS burst as measured by chemiluminescence (Figure 4B). The accumulation of ROS induced by PopW in TRV-*SlβCA1* leaves was significantly reduced. Callose accumulation was detected at 12 h after PopW treatment (Figure 4C). Moreover, the callose content in TRV-*SlβCA1* was significantly reduced (Figure 4D). Thus, we conclude that SlβCA1 plays a role in MAMP-activated defense responses.

### 2.5. Direct Physical Interaction of SlβCA1 with PopW

**Figure 5:**
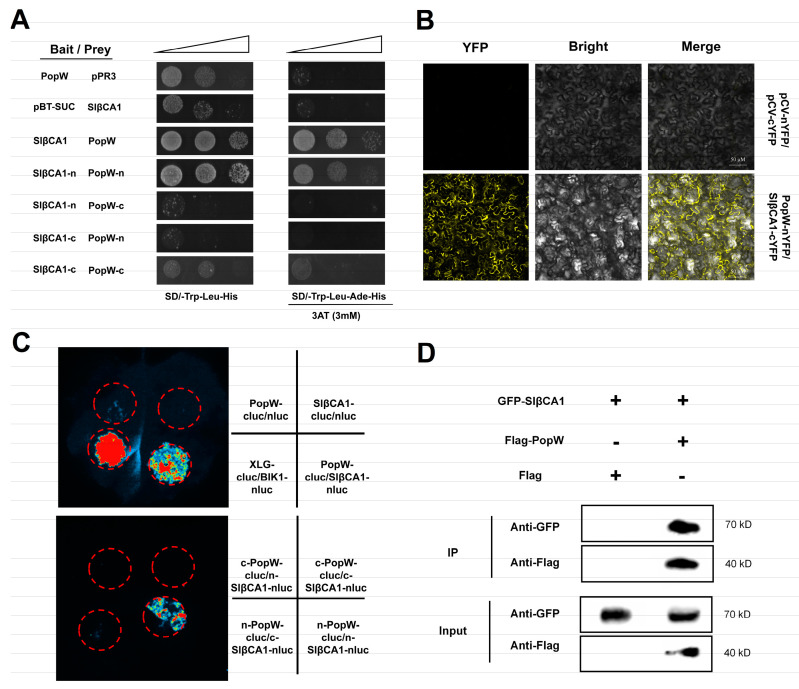
PopW and SlβCA1 interact directly. (**A**) Y2H. The interaction between pBT3-SUC-PopW and pPR3-SlβCA1 and the interaction between pBT3-SUC and pPR3 were used as negative controls,. pBT3-SUC-PopW and pPR3 and pPR3-SlβCA1 and pBT3-SUC showed self-activation. (**B**) BIFC. YFP fluorescence was observed by confocal microscopy. The interaction of nYFP and cYFP was used as a negative control. Scale bar = 50 μm. (**C**) LCA. The fluorescence signal intensity represents the interaction of two proteins. SlβCA1 and PopW interacted with a no-load on the other side, indicating self-activation. The interaction of nluc and cluc was used as a negative control. (**D**) COIP to test the SlβCA1-GFP and PopW-Flag interaction, using GFP-SlβCA1 and Flag as negative controls. Protein molecular weights: PopW = 39.79 kDa; SlβCA1 = 34.47 kDa.

Since SlβCA1 is involved in the PopW-mediated MAMP response, we hypothesized that they could interact directly. We first performed a yeast two-hybrid (Y2H) assay to verify the interaction and found that SlβCA1 interacted with PopW (Figure 5A). In the same bimolecular fluorescence complementation (BIFC) assay, yellow fluorescent protein (YFP) was introduced between PopW and SlβCA1. The signals indicated that they interacted on the membrane and nucleus (Figure 5B). Moreover, in the luciferase complementation assay (LCA), the luciferase signal was detected when SlβCA1 fused with one half of luciferase and PopW fused with the other half of luciferase were expressed in *N. benthamiana* (Figure 5C). Subsequently, we verified these results with coimmunoprecipitation (COIP). PopW tagged with Flag and SlβCA1 tagged with GFP were overexpressed in tobacco leaves, and Flag-empty was used as a control. The protein was enriched by anti-Flag magnetic beads. The results of antibody detection showed that GFP-SlβCA1 was present in the immune complexes precipitated by Flag-PopW, indicating that PopW and SlβCA1 interacted with each other (Figure 5D). In conclusion, these results indicate that PopW physically interacts with SlβCA1.

Next, to explore their interacting domains, we divided PopW into the N-terminal with a Harpin domain and the C-terminal pectate lyase (PL) domain, while SlβCA1 was divided into the C-terminus with a CA domain and the N-terminus. The interaction between the N-terminus of PopW with the Harpin domain and the N-terminus of SlβCA1 without the CA domain was demonstrated by the Y2H and LCA methods (Figure 5A,C).

### 2.6. SlβCA1 Is Involved in PopW-Induced Immunity to Xe 85-10

**Figure 6:**
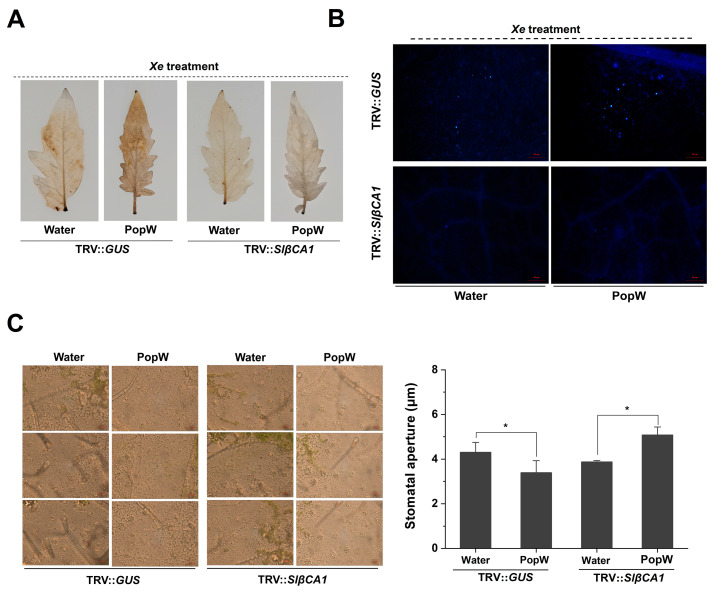
SlβCA1 supports PopW-induced immunity against *Xe* 85-10. (**A**–**C**) Samples inoculated with *Xe* 85-10 after treatment with PopW and water for 5 d were tested. (**A**) The ROS burst in tomato leaves after 3 h of *Xe* 85-10 treatment. (**B**) Callose accumulation in tomato leaves after 12 h of *Xe* 85-10 treatment. (**C**) Representative optical microscope images and quantitative measurements of pores in response to *Xe* 85-10. Measurements were performed 3 h after inoculation. The quantitative data for pore opening are shown here as a histogram. Scale bar = 20 μm. The asterisks indicate statistical significance by *t* test (* *p* < 0.05).

The N-terminal Harpin domain of PopW is involved in the regulation of the plant immune response [20]. To further investigate the role of SlβCA1 in PopW-induced resistance, we also examined PopW-induced ROS accumulation and callose staining during pathogen infection. In the PopW treatment, the accumulation of ROS and callose in TRV-*SlβCA1* was less than that in TRV-*GUS* (Figure 6A,B). 

**Figure 7:**
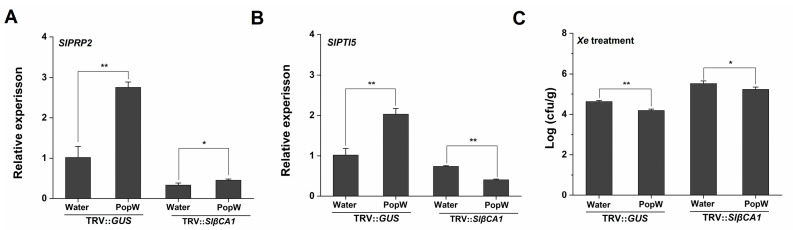
SlβCA1 can upregulate PopW-induced resistance genes in *Xe* 85-10 defense. (**A**,**B**) The expression levels of *SlPRP2* and *SlPTI5* in tomato leaves after 24 h of *Xe* 85-10 spraying treatment. The amount of the gene in the TRV-*GUS* sample was assigned a numerical value of 1. (**C**) Bacterial colonization on leaves. The measurement was carried out after 4 d of *Xe* 85-10 spraying treatment. The asterisks indicate statistical significance by *t* test (** *p* < 0.01; * *p* < 0.05).

Both PopW and CA have been reported to be involved in stomatal regulation [10,13,15,24]. Stomatal immunity is a typical defense response. We analyzed stomatal opening and closing in SlβCA1-silenced plants after treatment with *Xe* 85-10. In the PopW treatment group, the silencing of SlβCA1 significantly increased stomatal opening, which was more conducive to pathogen invasion (Figure 6C). This result was also consistent with the increased sensitivity of TRV-*SlβCA1* to pathogens (Figure 7C).

At the same time, in the PopW treatment group, silencing of SlβCA1 decreased the expression of the resistance genes (Figure 7A,B). In TRV-*SlβCA1*, the decrease in bacterial colonization due to PopW treatment was also suppressed (Figure 7C). Taken together, these results indicate that SlβCA1 modulates PopW-mediated immune responses.

### 2.7. The Involvement of SlβCA1 in Immunity Is Not Related to CA Activity

**Figure 8:**
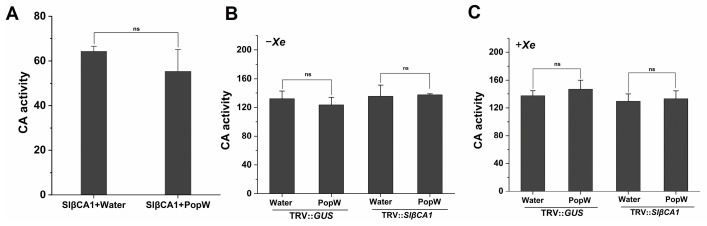
CA activity is not related to the immune function of SlβCA1. (**A**) Effect of PopW on the CA activity of recombinant His-SlβCA1 protein. (**B**) CA activity in leaves after PopW or water treatment for 12 h. (**C**) CA activity in leaves after inoculation with *Xe* 85-10 for 12 h. ns indicates no significant difference.

Structural analysis showed that SlβCA1 possesses a CA domain, so we sought to investigate whether SlβCA1-mediated responses to PopW and basal immunity to pathogens are related to CA activity. The recombinant SlβCA1 protein was incubated with PopW and treated with water as a control. The activity of the SlβCA1 protein incubated with PopW was not significantly different from that of the control (Figure 8A). 

We also tested CA activity in vivo. The results showed that there was no difference in CA activity between PopW-treated plants and the control (Figure 8B).

To further determine the relationship between the CA activity of SlβCA1 and the immunity it confers against pathogens, CA activity was measured. No significant difference was observed in CA activity among the treatment groups (Figure 8C). These results indicate that the CA activity of SlβCA1 is not involved in its role in the MAMP response and immune response.

## 3. Discussion

Tomato scab is a global bacterial disease caused by *Xanthomonas* sp. The pathogen can damage *Solanum lycopersicum*, *Capsicum* spp., *Physalis pubescens* and other *Solanaceae* Juss. plants, causing defoliation, stem cracking and scab on the fruit surface, resulting in serious yield loss and fruit quality decline [26,27,28,29]. Studies on the molecular mechanisms of plant resistance to this disease can provide a theoretical basis for disease control. *Xe* 85-10 is one of the pathogens causing tomato bacterial scabs. We identified SlβCA1 as a molecular regulatory switch that positively regulates resistance to *Xe* 85-10 in tomato plants.

An important function of βCA in plant metabolism is to catalyze the reversible hydration of CO_2_ to HCO_3_^−^ [9]. The enzyme activity of CA provides the substrate for the carboxylation (CO_2_) reaction of Rubisco in C_3_ photosynthesis [9]. Before this study, there was little information on the role of βCA in plant basal immunity in susceptible crop–pathogen interactions. Here, we provide evidence that SlβCA1 is activated in response to *Xe* 85-10 treatment and to increased inhibition of pathogen growth (Figure 1 and Figure 3). Our results demonstrate that the active role of SlβCA1 in plant basal immunity differs from that observed in the *Slβca3* mutant [19]. We hypothesize that activation of SlβCA1 leads to tomato innate immune responses. The following evidence supports this hypothesis. After inoculation with *Xe* 85-10, the transcript abundance of *SlβCA1* was greatly increased, while the transcript abundances of *SlβCA2*, *SlβCA3* and *SlβCA4* were unaltered or decreased (Figure 1). This finding is distinct from that observed when the plants were inoculated with *Pst* DC3000, which can only induce high expression of *SlβCA3* [19]. This suggests that tomato plants respond differently to different pathogens. Furthermore, *SlβCA1* activation is essential for immune response coordination. SlβCA1 is located in the nucleus, the membrane and the chloroplast. The biological function assay used in this study revealed that TRV-*SlβCA1* exhibited more severe disease symptoms and decreased expression of resistance genes after *Xe* 85-10 inoculation than TRV-*GUS* controls. These results support the conclusion that inducible SlβCA1-mediated defense pathways are essential for immunity to the virulent bacterial pathogen *Xe* 85-10 in tomato plants.

Previous studies have shown that βCA is responsive to effectors or elicitors. For example, NbβCA1 positively regulates the *Pto: AvrPto*-mediated hypersensitive defense response [15]. Additionally, in Arabidopsis, the expression of *AtβCA1* is required for complete defense against the avirulent bacterial pathogen *Pst* DC3000 carrying *avrB* [14]. In contrast, AtβCA1 and AtβCA4 play a negative role in the flg22-induced PTI response [10]. Our results suggest that *SlβCA1* gene expression is upregulated after PopW treatment and can positively regulate a series of PTI responses induced by PopW by four methods (Figure 5). At the same time, we verified that SlβCA1 is the interaction target of the elicitor PopW. Although it has been speculated previously that βCA can be used as a target of exogenous effectors [2], this is the first time this has been verified experimentally, which undoubtedly provides a new idea for the study of the role of βCA in immunity. Meanwhile, our results also show that SlβCA1 can positively regulate PopW-induced enhancement in *Xe* defense (Figure 6 and Figure 7). In addition, it seems likely that the enzymatic activity of CA has no relationship to its function in basal immunity (Figure 8).

Taken together, the data presented herein reveal a novel function of SlβCA1 activation in PopW-induced plant basal immune regulation. This information is very important for understanding the molecular mechanism of PopW-induced disease resistance in plants. Since orthologs of βCA have been identified throughout the plant kingdom, and it was found in our study that CA exhibits differential responses to different pathogens, it would be interesting to investigate whether βCAs are associated with differences in the underlying immunity to pathogens.

## 4. Materials and Methods

### 4.1. Plant Materials and Growth Conditions

Tomato and *N. benthamiana* were used as experimental materials. The tomato cultivar used was Hezuo 903, and the silenced tomato line was also derived from Hezuo 903. Both tobacco and tomato were grown in the same greenhouse. The light conditions were 18 h light and 6 h dark. The temperature during the day was 27 °C. The temperature in the night was 25 °C. The culture medium for tomato was vermiculite and nutrient soil (4:6), and the culture medium for tobacco was vermiculite.

The downregulation of gene expression in tomato was achieved by virus-induced gene silencing. First, as described online (https://sg.idtdna.com/Primerquest/Home/Index, accessed on 11 May 2022), *SlβCA1*-silenced fragments were selected from a tomato cDNA library. The fragment was cloned into the vector pTRV2 and transformed into *Agrobacterium tumefaciens* (GV3101) to silence the *SlβCA1* gene. Specific procedures were carried out by *Agrobacterium*-mediated transformation [19]. The primers used for construction and genotyping are listed in Supplementary Table S1. The silencing efficiency was measured at 6 weeks of age, and plants with silencing rates greater than 60% were selected for subsequent experiments.

### 4.2. PopW and Pathogen Acquisition and Disease Symptom Assay

Extraction and purification of PopW were carried out as described earlier [20]. *Xe* 85-10 carried a GFP-fluorescent marker and kanamycin-resistance selection marker, so it was cultured in Luria–Bertani (LB) medium containing 50 μg/mL kanamycin at 28 °C. Additional pathogens, including *Pst* DC3000 and *Rs* ZJ3721, were cultured in LB medium; *Pst* DC3000 was cultured at 28 °C, and *Rs* ZJ3721 was cultured at 37 °C. For pathogen treatment, the bacterial suspension cultured overnight was resuspended in pure water to OD_600_ = 0.3, and 0.02% Silwet L 77 was added, after which the mixture was sprayed on the leaves of 8-week-old tomato plants [24].

Disease status was assessed by quantifying bacterial leaf populations [24]. Bacterial quantitation was performed by the dilution point plate method. A total of 0.1 g of diseased leaves was ground, and 9 times the volume of water was added. The leaves were serially diluted (10^1^, 10^2^, 10^3^, 10^4^, 10^5^, 10^6^ and 10^7^) and mixed well. Then, 10 μL was used for spot plating, colonies were counted after 4 d, and the colony-forming unit (CFU) number was calculated [24].

### 4.3. Amino Acid Sequence Alignment and Tree Construction

Amino acid sequence alignment and tree construction were performed using the neighbor-joining method with MEGA-X. The SlβCA1 protein is highlighted in red. The sequences included SlβCA1 (Solyc02g086820), SlβCA2 (Solyc05g005490), SlβCA3 (Solyc02g067750), SlβCA4 (Solyc09g010970), NbβCA1.1 (Niben101Scf09253g00011.1), NbβCA1.2 (Niben101Scf06349g00008.1), NbβCA2.1 (Niben101Scf08637g01001.1), NbβCA2.2 (Niben101Scf01155g01009.1), NbβCA3.1 (Niben101Scf16378g01012.1), NbβCA3.2 (Niben101Scf03766g09001.1), NbβCA4.1 (Niben101Scf07739g00010.1), NbβCA4.2 (Niben101Scf13995g01012.1), AtβCA1 (AT3G01500), AtβCA2 (AT5G14740), AtβCA3 (AT1G23730), AtβCA4 (AT1G70410), AtβCA5 (AT4G33580) and AtβCA6 (AT1G58180).

### 4.4. Gene Expression Analysis by qRT-PCR

Measurement of gene expression was performed by using real-time fluorescence quantitative PCR with *SlUBI3* as an internal control [30]. Total RNA was extracted from tomato leaves and used as a template for quantification. The relative expression levels of *CAs* and resistance marker genes were determined.

### 4.5. Detection of the MAMP Response

#### 4.5.1. Measurement of ROS Production

The production of ROS was monitored in real time by chemiluminescence within half an hour after treatment [31], while DAB staining was used to detect the accumulation of ROS [32]. The chemiluminescence method was used with slight modifications. Leaves from 6-week-old tomato plants were punched for the experiments, and the working concentration of PopW was 0.1 mg/mL. In the DAB staining experiment, tomato leaves were stained after 3 h of treatment with *Xe* 85-10 or PopW.

#### 4.5.2. Detection of Callogens

The staining method for callogens detection was performed as described by Yuan et al. [33]. Tomato leaves treated with PopW or *Xe* 85-10 for 12 h were selected for the experiment and examined under an inverted microscope after decolorization.

### 4.6. Protein Interaction Analyses

The determination of protein interactions was performed by four methods, including a BIFC assay, COIP of plant protein extracts, a Y2H assay and an LCA. The specific methods were as follows:

In the COIP experiment, SlβCA1 was cloned into the pCAMBIA1300 vector with a GFP tag, and PopW was cloned into the pCAMBIA2300 vector with a Flag tag [34]. The pCAMBIA1300-SlβCA1 construct was also used in the subsequent subcellular localization experiment. For the specific method used for *Agrobacterium* transformation, please refer to the method of Seo et al. [35]. *Agrobacterium* carrying pCAMBIA1300-SlβCA1 and pCAMBIA2300-PopW was injected into tobacco leaves for 48 h, and total protein was extracted with lysate (Beyotime). For the next IP run, we used Flag beads to enrich Flag protein complexes (Beyotime). Detection of the total protein content (Input) and Flag protein complex (Output) was performed with GFP and Flag (Abmart, Nanjing Warbio Biotechnology Co., Ltd., Nanjing, China).

In the Y2H experiment, pBT3-SUC and pPR3 were used as bait and prey vectors, respectively [36]. The constructed vector was transformed into yeast NMY51, and the yeast cells were grown on auxotrophic media, including Trp-, His- and Leu-deficient medium (SD/Trp-Leu-His) and Trp-, His-, Ade- and Leu-deficient medium (SD/Trp-Leu-Ade-His), in which 3 mM 3AT was added to inhibit autoactivation.

In the BIFC experiment, pCV-C/N was used as the vector, with the C-terminus linked with PopW and the N-terminus linked with SlβCA1 [37]. *A. tumefaciens* strains carrying the pCV-C-PopW and pCV-N-SlβCA1 vectors were coinjected into tobacco. After 48 h, the tobacco leaves were placed under a laser confocal scanning microscope to observe the fluorescence status. If the target proteins interacted, the fluorescence signal of YFP appeared in the field of vision.

LCA was performed using the method of Zhou et al. [38]. We constructed two target proteins, pCAMBIA1300-NLuc (nluc) and pCAMBIA1300-CCLuc (cluc). If the two target proteins interacted, the NLuc and CLuc of the luciferase were spatially close and correctly assembled, thus exerting the luciferase activity, i.e., decomposing the substrate to generate fluorescence.

### 4.7. Detection of SlβCA1 Protein Localization

The specific method used for subcellular localization was as described by Zhang et al. [39]. In brief, SlβCA1 carrying GFP fluorescence was expressed in tobacco cells using a binary vector carried by *Agrobacterium*. We used DAPI staining as a marker for the localization of the nucleus and detected the autofluorescence of the chloroplast as a marker. The fluorescence of the final GFP fusion protein, DAPI fluorescence and autofluorescence of chlorophyll were observed together in tobacco leaves using a laser functional confocal microscope (Zeiss LSM 710, Oberkochen, Germany).

### 4.8. Determination of CA Activity

According to a previously described method by Hatch et al., CA was extracted from leaves [40]. Immediately after 12 h of treatment with PopW or *Xe* 85-10, approximately 0.1 g of fresh leaf tissue was ground in liquid nitrogen, and 1 mL of extraction buffer (phosphate-buffered saline (PBS), pH = 7.4) was added. The sample was centrifuged at 8000 rpm for 30 min, and the supernatant was examined. The determination of CA enzyme activity was performed with a Plant βCA Enzyme-Linked Immunosorbent Assay (ELISA) Kit (Shanghai Jingkang Biological Engineering Co., Ltd., Shanghai, China).

### 4.9. Measurement of Porosity

The method used for the determination of porosity was as previously described by Melotto et al. with slight modifications [41]. The method comprised the following steps: Tomato plants were placed under illumination conditions to ensure that the leaf stomata were in a completely open state. After the leaves were treated by *Xe* 85-10 for 3 h, epidermal tissues were removed with tweezers and placed under an inverted microscope to observe the stomatal apertures. Visual fields were randomly selected to measure the stomatal apertures, and at least n ≥ 100 stomata were measured in each treatment.

### 4.10. Statistical Analysis of the Results

All experiments were performed in triplicate, and the values in the figures represent the mean ± SD of three replicates [42]. Asterisks indicate significant differences between data sets (* indicates 0.01 < *p* < 0.05; ** indicates *p* < 0.01). Orign 2018 was used for data processing and mapping.

## 5. Conclusions

In this study, SlβCA1 was the main target and was found to be localized in the cell membrane, nucleus and chloroplast. *SlβCA1* could respond to the treatment with the tomato scab pathogen *Xe* 85-10, so SlβCA1 may participate in the immune response of tomato to *Xe* 85-10. First, we observed that the *Xe* 85-10 colonization level in TRV-*SlβCA1* increased, and the expression of resistance genes was inhibited. In addition, the expression of *SlβCA1* was also induced by PopW. We systematically analyzed the PopW-induced MAMP response and found that SlβCA1 could inhibit PopW-induced ROS burst and callose accumulation. PopW and SlβCA1 could directly interact. The disease resistance of tomato against *Xe* 85-10 induced by PopW was affected, including via ROS burst, the accumulation of callose, reduction in the colonization level, upregulation of disease-resistance genes and closure of stomata. The CA activity of SlβCA itself was not involved in these immune processes. Our data highlight the important role played by SlβCA1 in basic plant immunity, supporting further research on the function of CA in immunity. The mechanism by which CA participates in surface recognition and interacts with PopW during in vivo interactions between plants and pathogens and, crucially, the identification of CA as a plant cell surface receptor were reported here for the first time, providing novel insights into the direction of the interactions between plants and pathogens.

## Data Availability

The data that support the findings of this study are available from the corresponding author upon reasonable request.

## Supplementary Materials

The supporting information can be downloaded at https://www.mdpi.com/article/10.3390/ijms241311021/s1.
